# Application of multi-omics in the study of traditional Chinese medicine

**DOI:** 10.3389/fphar.2024.1431862

**Published:** 2024-09-06

**Authors:** Meng Zhao, Yanan Che, Yan Gao, Xiangyang Zhang

**Affiliations:** School of Pharmaceutical Science and Technology, Tianjin University, Tianjin, China

**Keywords:** traditional Chinese medicine, mass spectrometry, proteomics, metabolomics, mass spectrometry imaging

## Abstract

Traditional Chinese medicine (TCM) is playing an increasingly important role in disease treatment due to the advantages of multi-target, multi-pathway mechanisms, low adverse reactions and cost-effectiveness. However, the complexity of TCM system poses challenges for research. In recent years, there has been a surge in the application of multi-omics integrated research to explore the active components and treatment mechanisms of TCM from various perspectives, which aids in advancing TCM’s integration into clinical practice and holds immense importance in promoting modernization. In this review, we discuss the application of proteomics, metabolomics, and mass spectrometry imaging in the study of composition, quality evaluation, target identification, and mechanism of action of TCM based on existing literature. We focus on the workflows and applications of multi-omics based on mass spectrometry in the research of TCM. Additionally, potential research ideas for future exploration in TCM are outlined. Overall, we emphasize the advantages and prospects of multi-omics based on mass spectrometry in the study of the substance basis and mechanism of action of TCM. This synthesis of methodologies holds promise for enhancing our understanding of TCM and driving its further integration into contemporary medical practices.

## 1 Introduction

Traditional Chinese medicine (TCM) has been inherited and developed over thousands of years. It offers unique insights into disease treatment, emphasizing syndrome differentiation to target various organ systems in the human body through multiple links, pathways, and targets (Tian et al., 2022; Zheng et al., 2020). This dialectical treatment mechanism is consistent with the complex pathological mechanisms involved in diseases, allowing for comprehensive regulation of the human body from a holistic perspective to effectively prevent and treat diseases. While chemical drugs currently dominate disease treatment, they often lead to various side effects and adverse reactions, potentially resulting in drug resistance after prolonged use (Artasensi et al., 2020). In contrast, TCM boasts distinct advantages in disease prevention and treatment, characterized by precise therapeutic effects, minimal adverse reactions, and high cost-effectiveness. Consequently, TCM has garnered significant attention globally and is increasingly integrated into disease management practices worldwide (Liu et al., 2013). Presently, global research on TCM can be broadly categorized into four main aspects: (1) Screening: involves identifying potentially active TCM based on their properties, comparison with the treatment mechanisms of chemical drugs, as well as pharmacological assessments; (2) Confirmation: entails validating the efficacy of TCM through animal experiments or clinical trials; (3) Exploration: focuses on elucidating the mechanism of action and effective components of active TCM; and (4) Application: emphasizes the utilization of TCM in clinical settings for enhanced outcomes (Wang et al., 2022).

Multi-omics is a series of research methods based on high-throughput analysis and detection techniques in the modern biological research system, including genomics, transcriptomics, proteomics and metabolomics, etc. (Le et al., 2016). These techniques facilitate a comprehensive understanding of molecular complexities at different levels, aiding in the exploration of the intricate relationships between human health and diseases. TCM has the characteristics of complex components and diverse targets, the conventional single pathway studies may struggle to interpret the therapeutic concept of holistic concept (Li et al., 2015). Multi-omics have characteristics such as holism, dynamism, spatiotemporal variability, and complexity, which align well with this holistic concept. While multi-omics has significant advantages in TCM research, there are also some limitations. In Supplementary Table S1, the advantages and disadvantages of multi-omics are summarized. With the completion of the Human Genome Project, simple genome research can no longer meet the needs of scientific research, and the focus of life sciences has shifted to the study of gene expression products and proteins. Compared to genes and RNA, proteins are the ultimate carriers of biological functions, and the proteome represents the state in which organisms perform direct functions, reflecting the true expression of gene transcription (Guo et al., 2023). Metabolites are the downstream of transcriptional expression networks and protein interaction networks, which can reflect changes in the phenotypic state of organisms and explore the metabolic mechanisms of the entire organism. Therefore, proteomics and metabolomics have unique advantages in the field of TCM research, as they can discover information that other omics cannot. In order to comprehensively elucidate the spatial and temporal changes of natural components and metabolites, it is necessary to use spatial omics, which can provide deeper insights into pathological biological processes. Mass spectrometry imaging (MSI) has demonstrated its advantages in analyzing molecules (proteins, peptides, lipids, metabolites, etc.) in complex biological samples at the spatial resolution level, which makes it possible to understand the position of molecules in tissues, indicate changes in their expression in time and space, and help identify unknown cellular regulatory processes (Wu et al., 2022; Ma and Fernández, 2022; Holzlechner et al., 2019; von Eggeling and Hoffmann, 2020; Cilento et al., 2019).

In recent years, multidimensional studies on signaling pathways, genes, cells, animal experiments, and clinical trials have convincingly highlighted the therapeutic benefits of TCM in disease management and prevention. The integration of multi-omics research allows for a comprehensive exploration of TCM’s mechanisms from diverse perspectives, thereby facilitating the broader application of TCM in clinical practice and contributing significantly to the modernization of TCM (Le et al., 2016; Wang et al., 2013a). In this review, we provide a summary of workflows and application of mass spectrometry based proteomics, metabolomics, and MSI in the study of TCM. Additionally, we highlight the potential research ideas and combination of multi-omics for future exploration in TCM.

## 2 Analytical techniques

The rapid development of analytical instruments is accelerating research on the multi-omics of TCM (Lao et al., 2009). Mass spectrometry (MS) is a technique that employs various ionization methods to convert substance molecules into ions, which are then separated and measured based on their mass-to-charge ratio (m/z) differences for the analysis of substance composition and structure. Under vacuum conditions, vapor molecules in gaseous, solid, or liquid states lose their external valence electrons and form positively charged molecular cations. If the energy exceeds the required ionization threshold, specific chemical bonds within molecular ions may further break, giving rise to fragmented ions of varying masses (Loos et al., 2016; Gad et al., 2013; Dharmaraj et al., 2006). Nuclear magnetic resonance (NMR) serves as a non-selective universal analysis method capable of analyzing nearly all hydrogen-containing compounds. However, the components detected primarily consist of high-concentration substances with low sensitivity (Li et al., 2015). In addition, there are other techniques such as Liquid Chromatography (LC) (Yang et al., 2004; Yang et al., 2005; Pham-Tuan et al., 2003), Gas Chromatography (GC) (Chace and Kalas, 2005), Capillary Electrophoresis (CE) (Fan et al., 2012), Ultraviolet-visible spectroscopy (UV-Vis) (Vlachos et al., 2006), and Fourier Transform Infrared spectroscopy (FTIR) (Kokalj et al., 2011; Ronda et al., 2008). These techniques have their own advantages and disadvantages, as shown in Supplementary Table S2.

In the research of TCM, due to the complex components, multiple analytical techniques are typically integrated and combined. For example, gas chromatography-mass spectrometry (GC-MS) technology, known for its high sensitivity, is well-suited for comparative studies of volatile oil components in TCM (Li et al., 2015). Liquid chromatography-mass spectrometry (LC-MS) and liquid chromatography with tandem mass spectrometry (LC-MS/MS) involve the chromatographic separation of analytes followed by the detection of their m/z values. By leveraging high-performance liquid chromatography (HPLC) or ultra-performance liquid chromatography (UPLC), the complexity of compounds extracted from samples can be significantly reduced, allowing for the detection of a broader range of compounds through MS (Hou et al., 2023).

## 3 Proteomics in the study of TCM

Proteomics involves conducting large-scale research on proteins through biochemical methods, which bridges the gap between TCM and modern medicine, offering valuable insights into disease treatment mechanisms, objective evaluation systems, and new drug development (Xin et al., 2018). Using proteomics as a foundation for TCM research offers clear advantages in modern experimental studies. This is primarily evident in how proteomics approaches the targets and mechanisms of TCM from a holistic viewpoint by comparing differential protein expression in control group cells or animal tissues with post-administration group of TCM. This approach aids in uncovering the molecular mechanisms of TCM in disease treatment, establishing an objective evaluation system, and providing new ideas and methodologies for the development of new drugs (Zhu et al., 2016).

### 3.1 Workflow of proteomics in TCM

**FIGURE 1:**
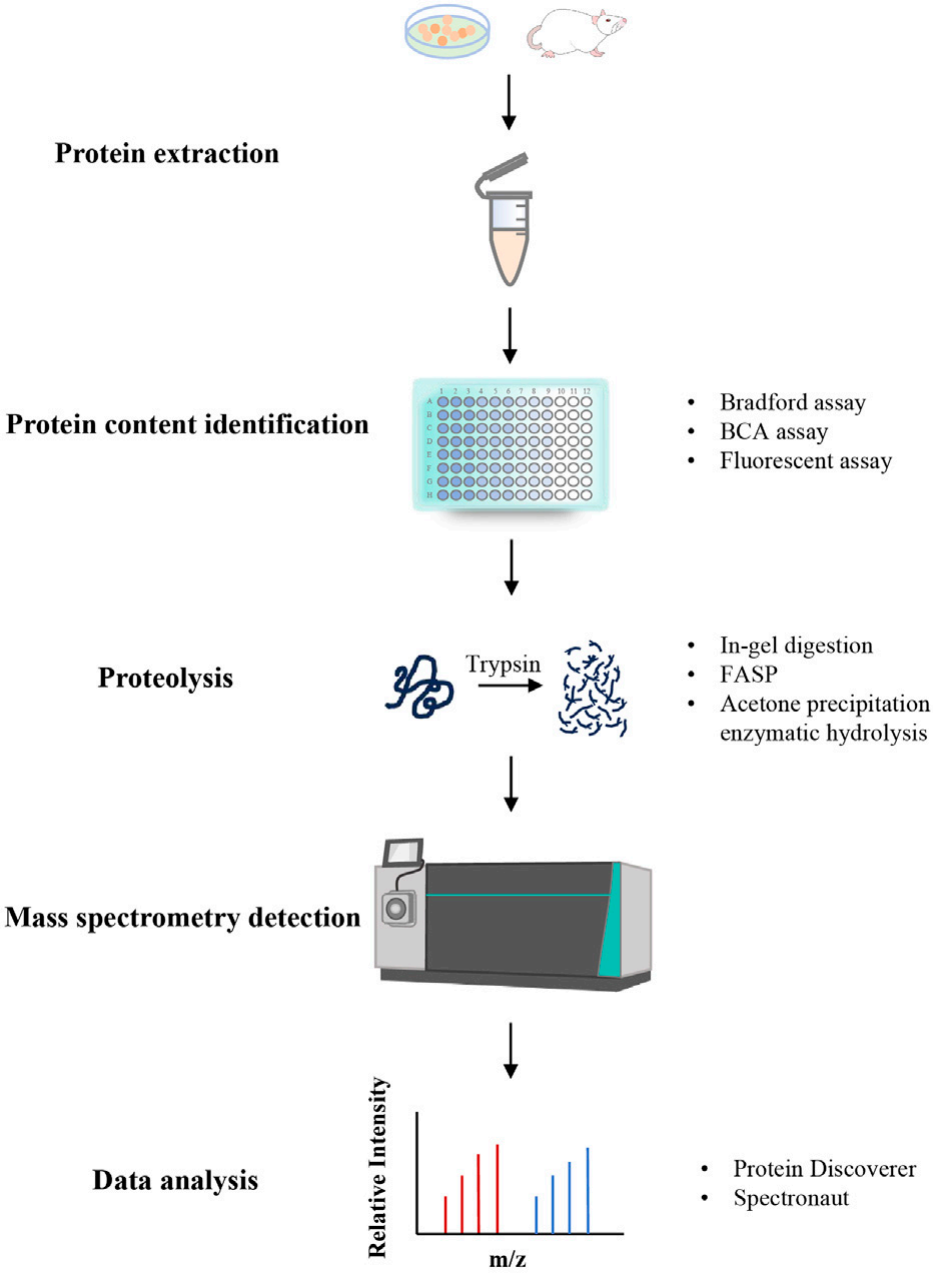
The workflow of proteomics.

In recent years, the continuous development of proteomics and high-resolution MS technology has provided new ideas and technical support for establishing high-throughput research methods for protein components and conducting associated investigations. Presently, proteomics researches on proteins or peptides mainly adopts Shotgun strategy based on the Bottom-up MS identification method, which involves separating and purifying proteins or peptides for high-resolution MS analysis to acquire MS2 data. Subsequently, database retrieval software such as Mascot and SEQUEST can be used to accomplish the identification of proteins or peptides (Xin et al., 2018; Yang et al., 2019). Establishing a disease model, administering TCM treatment, and then examining its mechanism of action via proteomics is a common research approach in TCM proteomics (Wang, 2019). The fundamental workflow of proteomics is shown in Figure 1.

#### 3.1.1 Protein extraction

For protein extraction in disease models post-administration, tissue, serum, or urine are typically utilized as samples. Taking tissues as an example, animal tissues are initially collected and then homogenized by adding an appropriate quantity of lysis buffer solution, such as Urea lysis solution, Sodium Dodecyl Sulfate (SDS) lysis buffer, phosphate-buffered solution (PBS), and Radio Immunoprecipitation Assay (RIPA) lysis buffer (Yin et al., 2019). Subsequently, cell membrane disruption is conducted through ultrasound within the lysis solution environment. It is important to include protease inhibitors like Phenylmethanesulfonylfluoride (PMSF), Ethylenediaminetetraacetic Acid (EDTA), Cocktail, and phosphatase inhibitors such as Sodium Fluoride, Sodium Orthovanadate, Sodium Pyrophosphate, and β-Glyphophosphate in the lysis buffer solution (Konno et al., 2024).

#### 3.1.2 Protein content determination

For the determination of protein content, Bradford assay, Bicinchoninic acid (BCA) assay, and fluorescent assay are commonly used. In the Bradford assay, basic amino acids (particularly arginine) and aromatic amino acid residues from proteins bind to Coomassie dye G-25 under acidic conditions, resulting in a color shift from brown to blue. This method requires the measurement of absorbance at 595 nm. In contrast, the BCA assay involves proteins reducing Cu^2+^ to Cu^+^ under alkaline conditions, where Cu^+^ forms a purple complex with the BCA reagent. Absorbance is measured at 562 nm. The fluorescent assay is typically utilized to determine the tryptophan content in a sample, divided by 1.3% to determine the protein content. However, it is unsuitable for samples like serum and urine (Ren, 2014).

#### 3.1.3 Proteolysis

Currently, three widely used methods for proteolysis exist, with Trypsin being the more commonly used enzyme. The first method is In-gel digestion, where Sodium Dodecyl Sulfate-Polyacrylamide Gel Electrophoresis (SDS-PAGE) gels are typically cut into small pieces. Enzymes are added to the Ammonium Bicarbonate solution system for proteolysis after staining, reducing, and alkylating within the gel. Subsequently, peptides generated from the gel are extracted. The second method is Filter Aided Sample Preparation (FASP), which involves using a membrane device for solution replacement. Urea effectively eliminates detergents from the solution system, replacing them with either Ammonium Bicarbonate or Tetraethylammonium Bromide (TEAB) for protein digestion. Upon completion of proteolysis, peptides are collected from the flow-through components of the membrane. In acetone precipitation enzymatic hydrolysis, 4–6 times volume of cold acetone is usually added for protein precipitation. Urea aids in redissolving the protein easily, followed by enzyme addition for proteolysis. Once the proteolysis concludes, the system must be acidified to halt the enzymatic hydrolysis reaction (Hu et al., 2022).

#### 3.1.4 Mass spectrometry detection and data analysis

MS stands as the most frequently utilized technology for protein identification. As an essential technical platform for proteomics research, it is currently the fastest developing, most dynamic and potential technology in proteomics. Ion sources, mass analyzers, and detectors are the core components of mass spectrometers. Ion sources used for protein samples mainly consist of matrix-assisted laser desorption ionization (MALDI) and electrospray ionization (ESI). Commonly used mass analyzers include Fourier transform ion cyclotron resonance (FTICR/FT), linear ion trap (LIT/LTQ), quadrupole ion trap (QIT), time-of-flight (TOF), and so on. There are also various MS detectors, including electron multiplier tubes, ion counters and induced charge detectors (Buriani et al., 2012). In the study of proteomics, the “soft ionization” method is frequently applied to ionize protein molecules without forming fragment ions, thereby preserving the integrity of the entire protein molecule. Before detecting the obtained peptides by MS, desalination of peptide segments is essential. Subsequently, the generated spectra are matched with theoretical spectra corresponding to the sample using LC-MS/MS and data-independent analysis (DIA) collection modes to determine the amino acid composition of the peptides, so that the protein can be inferred based on the peptides (Li et al., 2013). In general, the more actual spectra that can be matched, the more reliable the protein identification outcomes. Subsequent data processing often involves multivariate statistical analysis in combination with chemometrics, with commonly used software including Protein Discoverer, Spectronaut, etc.

### 3.2 Application of proteomics in TCM

With technological advancements, the application of proteomics in TCM scientific research is growing, expanding its scope to encompass the study of targets and mechanisms of action of TCM. Proteomics has already yielded significant results in TCM research and is currently in a stage of continuous development and gradual improvement. Proteomics is gradually merging with genomics, transcriptomics, and metabolomics, paving the way for a comprehensive understanding of TCM and catalyzing the rapid advancement of TCM research. Here, we summarize proteomics studies of TCM and highlight the application of proteomics in action targets and mechanism of action of TCM, as shown in Table 1.

**TABLE 1:** The application of proteomics in action targets and mechanism of action of TCM.

Application	Study findings	Reference
Action targets	21 differentially expressed proteins that could serve as potential targets for the toxic effect of triterpenoid compound Ganoderic acid D on human cervical cancer cell line Hela were detected	Yue et al. (2018)
	The TCM syndrome-specific DEPs were confirmed as biomarkers of HIV/AIDS, including FN1, GPX3, KRT10 for AHT and RBP4, ApoE, KNG1 for YDSK	Wen et al. (2018)
	STMN1 may be a major target for gambogic acid (GA) and gambogenic acid (GEA) in combating hepatocellular carcinomae (HCC)	Wang et al. (2019)
	BYHW can reduce the neurological impairments focus on IL-1β, IL-6, TNF-α, MCP-1, MMP-9, and PAI-1	Li et al. (2023)
Mechanism of action	Effects on migraine patients with hyperactivity of liver-yang syndrome may be related to regulating the blood lymphocyte protein expression	Zhong et al. (2009)
	Mechanism of Biyuan Tongqiao Granules in treating allergic rhinitis may be related to regulating PI3K/AKT and STAT3/MAPK signaling pathways	Wang et al. (2024)
	Ganshu Nuodan can protect acute alcoholic induced liver injury by inhibiting oxidative stress, lipid accumulation and apoptosis	Yang et al. (2023)
	The YJMP effect of PG may be directly associated with the Golgi-ER transport system	Liang et al. (2024)

#### 3.2.1 Application of proteomics in action targets of TCM

By comparing the protein expression spectra of control cells or animal tissues with those post-administration of TCM, potential target-related proteins of TCM can be identified. Moreover, through proteomics, the effective targets of active ingredients in TCM can be identified, aiding in guiding and predicting the discovery and isolation of chemical components in TCM. For instance, Yue et al. investigated the mechanism of Ganoderic acid D, a triterpenoid compound in Ganoderma lucidum, on the cytotoxicity of the human cervical cancer cell line Hela using two-dimensional electrophoresis combined with matrix-assisted laser desorption ionization mass spectrometry (MALDI-MS) and electrospray ionization mass spectrometry (ESI-MS). They detected 21 differentially expressed proteins that could serve as potential targets and constructed a network diagram of protein-protein interactions (PPI) (Yue et al., 2018). Wen et al. conducted proteomic comparative analysis on clinical cases of prevalent human immunodeficiency virus infection and acquired immune deficiency syndrome (HIV/AIDS) with TCM syndromes: accumulation of heat-toxicity (AHT) and Yang deficiency of spleen and kidney (YDSK). It was found that TCM syndrome specific differentially expressed proteins (DEPs) including FN1, GPX3, KRT10 for AHT and RBP4, ApoE, KNG1 for YDSK were confirmed as biomarkers. Thus, they provided a clinical and biological basis for the differential diagnosis of TCM syndromes of AIDS (Wen et al., 2018). Wang et al. utilized proteomics to explore the inhibitory effect of S-gambogic acid on the proliferation of the human liver cancer cell line HepG2 through the molecular target statin (Wang et al., 2019). Li et al. explored the neuroprotective effect and potential target protein of Buyang Huanwu Decoction (BYHW) in cerebral infarction (CI). They aluated the efficacy by TCM syndrome score and clinical indicators, and explored the changes of serum proteins by proteomics, so as to explore potential target proteins. The study found that BYHW can reduce the neurological impairments focus on IL-1β, IL-6, TNF-α, MCP-1, MMP-9, and PAI-1 (Li et al., 2023).

#### 3.2.2 Application of proteomics in mechanism of action of TCM

The process of TCM action is complex, and its underlying mechanism remains unclear. Through proteomics, it becomes feasible to directly compare the effects and alterations induced by TCM or compound formulations on the quantity and types of proteins during treatment. It aids in exploring the targets and mechanisms of TCM action while unveiling the interaction mechanisms between drugs and the body. Additionally, effective components and synergistic relationships can be discovered, laying a solid foundation for new drug development. Zhong et al. researched pathogenic mechanisms in migraine with hyperactive liver-yang syndrome and the curative mechanisms of calming liver and suppressing liver-yang treatment by collecting 32 migraine patients with hyperactivity of liver-yang syndrome. The proteomics results showed that the effects on migraine patients with hyperactivity of liver-yang syndrome may be related to regulating the blood lymphocyte protein expression (Zhong et al., 2009). Wang et al. combined network pharmacology and proteomics to elucidate the mechanism and targets of Biyuan Tongqiao Granules in treating ovalbumin-induced allergic rhinitis in mice. The result suggested that it may be related to regulating PI3K/AKT and STAT3/MAPK signaling pathways (Wang et al., 2024). Yang et al. provided a scientific basis for the hepatoprotective effect of Ganshu Nuodan in acute ALD mice and supported its traditional application. They identified three signaling pathways through proteomics which protect acute alcoholic induced liver injury in mice, namely Arachidonic acid metabolism, Retinol metabolism, and HIF-1 signaling pathway. It is shown that Ganshu Nuodan can protect acute alcoholic induced liver injury in mice by inhibiting oxidative stress, lipid accumulation and apoptosis (Yang et al., 2023). Liang et al. employed modern chemical and molecular biology methods to confirm the “Yin-Jing” effect of Platycodon grandiflorum (PG), and further revealed the “Yin-Jing” mechanism of action by proteomics. They speculated that the YJMP effect of PG may be directly associated with the Golgi-ER transport system (Liang et al., 2024).

### 3.3 Research ideas of proteomics in TCM

Applying proteomics to TCM research, potential target-related proteins of TCM can be identified by comparing differential protein expression in control group cells or animal tissues with post-administration group of TCM. Proteomics in TCM primarily aims to explore active proteins associated with drug efficacy, discover and confirm drug targets, reveal the molecular mechanisms of changes in secondary metabolites in TCM, and understand their effects on disease treatment. For research ideas of proteomics in TCM, the following points can be considered: (1) Comparing proteomics expression variances between normal and diseased groups, as well as those treated with TCM monomers, single drugs, and compound extracts, allows for the identification of potential target proteins. (2) Utilizing bioinformatics to predict interactions between TCM monomer compounds and specific targets, followed by screening and validating *in vitro* interactions of TCM monomer compounds. (3) Comparing proteomics results and integrating protein information from interactions with TCM in protein interaction databases, bioinformatics can be used to construct protein interaction networks associated with TCM interactions. Biological experimental techniques like yeast two-hybrid, immunoprecipitation, gene overexpression, and gene knockout can be utilized to validate protein interaction signal networks for specific targets. (4) Comparing the protein modification of TCM after disease treatment, such as histone acetylation and ubiquitination modifications, enables to investigate the modification effects of TCM on target proteins (Le et al., 2016; Wang, 2019).

## 4 Metabolomics in the study of TCM

Metabolomics is a novel technology that has swiftly evolved following genomics, transcriptomics, and proteomics. It possesses holistic and systematic characteristics that align with the concept of syndrome differentiation and a comprehensive view in TCM. Metabolomics holds promising applications in TCM research by leveraging high-quality MS data acquisition along with various data processing methods to extract biologically relevant information for experimental objectives (Tang et al., 2021). We believe that with the further development of metabolomics, particularly in multivariate analysis technologies, it will significantly advance TCM research, support the modernization of TCM, enhance the safety of applying modern methodologies in TCM evaluation, aid in establishing safety standards for TCM, and contribute to the formulation of international standards.

### 4.1 Workflow of metabolomics in TCM

**FIGURE 2:**
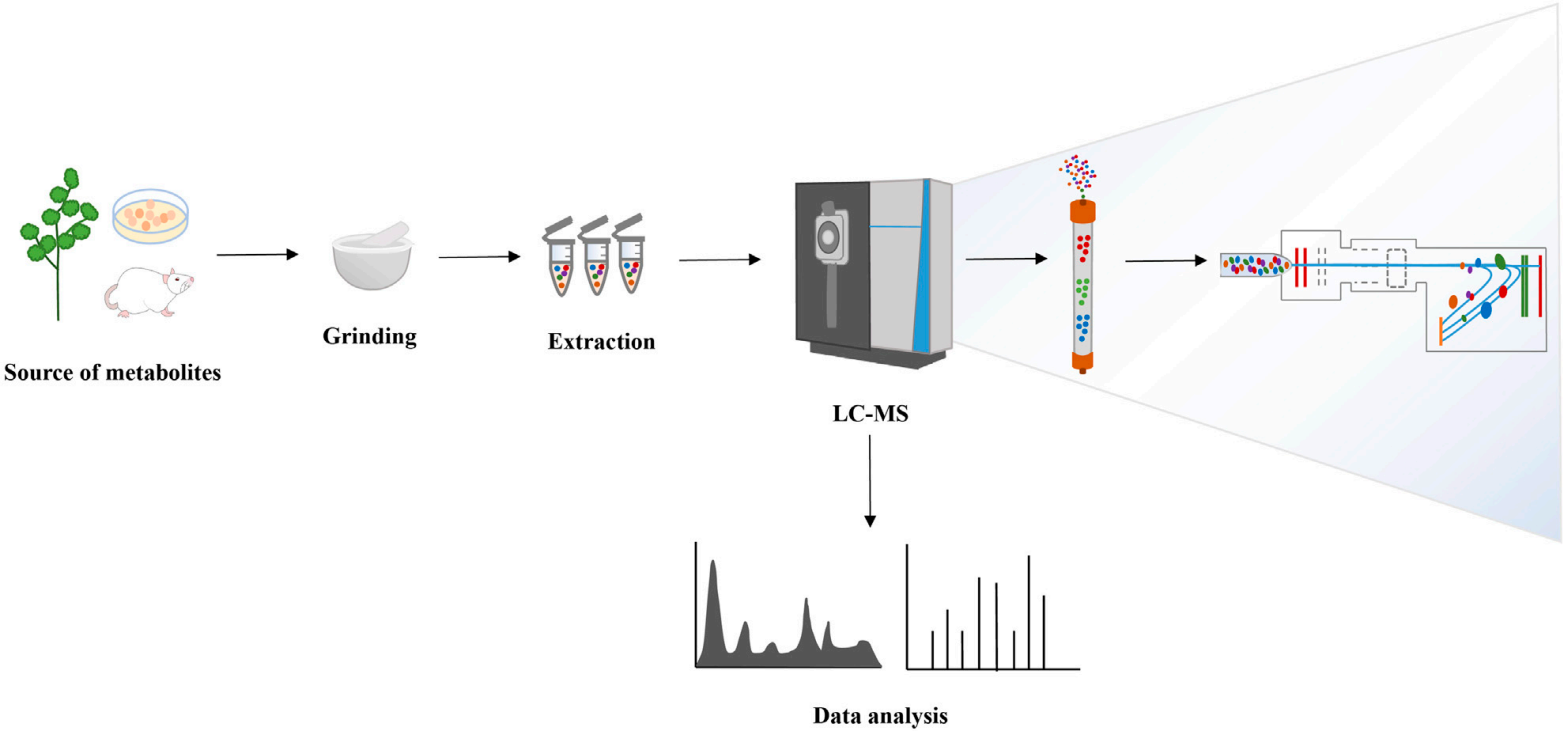
The workflow of metabolomics.

The detection of small molecule metabolites in organisms presents significant challenges due to their diverse chemical structures, substantial content variations, and complex physicochemical properties. Major platforms for metabolite identification involve MS and NMR. While NMR offers simplicity in sample preparation, high reproducibility, and excellent detection objectivity, MS boasts high resolution and sensitivity, making it particularly suitable for complex samples such as plants (Duan et al., 2016). The continuous development and advancements in MS technology enable the accurate detection and identification of an increasing number of metabolites in organisms, significantly propelling metabolomics research forward. Chromatography exhibits robust separation capabilities. The fusion of LC and MS enhances the sensitivity and resolution of metabolite identification and quantification, offering versatility in analyzing multiple metabolites (Tang et al., 2021). Moreover, current technological platforms for metabolomics research include GC-MS, capillary electrophoresis-mass spectrometry (CE-MS) (Barbas et al., 2011), and inductively coupled plasma-mass spectrometry (ICP-MS) (Coetzee et al., 2014), etc. Presently, LC-MS stands as the primary detection method for analyzing metabolomics samples (Carrola et al., 2018). In recent years, researchers have continually refined metabolite identification algorithms to enhance their metabolite recognition abilities (Shen et al., 2019). Concurrently, research strategies are evolving from static metabolite assessments to dynamic organismal changes monitoring. Isotope labeling-based metabolite tracking technology permits real-time monitoring of metabolic dynamics *in vivo*, facilitating a comprehensive understanding of metabolic processes within organisms (Jang et al., 2018). The fundamental workflow of metabolomics is shown in Figure 2.

#### 4.1.1 Sample pretreatment

Reflux and ultrasonication are commonly used for plant sample extraction. Water serves as the preferred solvent for polar compounds, while alcohol is utilized for compounds with lower polarity. When extracting metabolites from cells or animal models post-treatment, tissues, urine, and serum typically serve as the primary samples. For instance, when working with tissue samples, animal tissue is initially sectioned into pieces. Subsequently, pre-cooled solvent is added, followed by homogenization in a tissue grinder. The process involves incubation, centrifugation, vacuum drying, reconstruction, and other steps to prepare the test sample before analysis.

#### 4.1.2 LC-MS detection and data processing

MS enables the identification and quantification of metabolites by examining their chemical structures and differences in MS signal intensity. Reverse-phase chromatography, a component of chromatography, consists of a non-polar stationary phase and a polar mobile phase, representing the most prevalent separation technique. Metabolites identification is necessary to draw biological conclusions from metabolomics data. Analytes identification can be performed by searching the experimental data through databases available to the public for free (e.g., ChemSpider, METLIN, Human Metabolome DataBase, MassBank, mzCloud, GNPS, and LipidBlast), or for a nominal fee (e.g., NIST Mass Spectral Library). There are five levels of confidence in metabolites identification, namely validated identification (Level 1), putative identification (Level 2), preliminary identification (Level 3), molecular formula candidates (Level 4), and deconvolved experimental m/z (Level 5) (Schrimpe-Rutledge et al., 2016; Aharoni et al., 2023). Confidence annotation and statistical evaluation approach are shown in Supplementary Table S3. Subsequently, multivariate statistical analysis in conjunction with chemometrics methods aids in processing data to extract valuable insights from extensive datasets. Data processing involves key preprocessing steps like peak extraction, alignment, normalization, etc. These steps can be efficiently executed using specialized online data analysis platforms or proprietary software developed by instrument manufacturers such as MetaboAnalyst, Agilent Mass Hunter, Waters Progression QI and Compound Discoverer (Tang et al., 2021; Li et al., 2018; Forsberg et al., 2018).

### 4.2 Application of metabolomics in TCM

In recent years, metabolomics has assumed an increasingly important role in exploring the chemical components, quality evaluation and mechanism of TCM. It presents broad application and development prospects in clinical medicine, contributing significantly to biomarker discovery in clinical diagnosis, exploration and application of disease etiology and pathogenesis, and guiding clinical medication practices (Ning et al., 2013; Zhang and Shen, 2018). Here, we summarize metabolomics studies of TCM and highlight the application of metabolomics in components, quality evaluation and mechanism of action of TCM, as shown in Table 2.

**TABLE 2:** The application of metabolomics in components, quality evaluation and mechanism of action of TCM.

Application	Study findings	Reference
Components	Totally 1,100 metabolites belonged to 18 categories of E. ulmoides leaves of 193 core collections were comprehensively characterized	Meng et al. (2023)
	Chemical component correlations and disparities in rubber medicinal materials were explored via metabolomics by UPLC-MS	Su et al. (2021)
	Chemical composition of raw and processed Euphorbia pekinensis (EP) were analyzed and compared	Liu et al. (2024a)
Quality evaluation	A total of 10 quality disparity markers across different licorice processing techniques were identified	Wen et al. (2020)
	Quality markers were discovered in JGCC, such as abrine, trigonelline, hypaphorine and isoschaftoside	He et al. (2023)
	11 potential markers were selected to assess the quality of Aurantii Fructus and he best harvest period was within 1 week before and after the Lesser Heat	Liu et al. (2024b)
Mechanism of action	The mechanism of action of Xuefu Zhuyu Tang in treating coronary heart disease may be related to the processes of bile acid metabolism, lipid metabolism, and linoleic acid metabolism	Yi et al. (2019)
	CNAG with GHSC patients were associated with disturbed branched-chain amino acid metabolism and glycerophospholipid levels	Wen et al. (2024)
	The mechanism by which HSSD enhances the immunogenicity of CVS vaccine may be related to differential regulation of purine metabolism, vitamin B6 metabolism, folate biosynthesis, arginine and proline metabolism, and steroid hormone biosynthesis	Tang et al. (2024)

#### 4.2.1 Application of metabolomics in components of TCM

Metabolomics facilitates the high-throughput determination of plant metabolites, offering an objective perspective on the chemical composition variances in TCM that reflect differences in medicinal efficacy. Meng et al. used GC-MS and LC-MS/MS based non-targeted metabolomics comprehensively characterized metabolites profiles of E. ulmoides leaves of 193 core collections. Totally 1,100 metabolites belonged to 18 categories were identified, which contained 120 active ingredients for TCM and 85 disease-resistant metabolites (Meng et al., 2023). Additionally, Su et al. explored the chemical component correlations and disparities in rubber medicinal materials via metabolomics by UPLC-MS (Su et al., 2021). Furthermore, Liu et al. employed a combination of metabolomics and multivariate statistical analysis to analyze and compare the chemical composition of raw and processed Euphorbia pekinensis (EP). In total, 47 components were identified from the PEP samples in both positive and negative ionization modes, primarily belonging to flavonoids, terpenoids, organic acids, glycosides, and fatty acids. Among the raw EP group and PEP S4 group, 10 differential compounds were identified (Liu B. et al., 2024).

#### 4.2.2 Application of metabolomics in quality evaluation of TCM

The distinctive metabolite profiles among various origins and types of medicinal materials can be elucidated through metabolomics. Subsequently, by analyzing the metabolomics data alongside multivariate statistical methods, it becomes feasible to differentiate and pinpoint the varieties and origins of TCM, thereby establishing quality assessment criteria for different TCM varieties (Tang et al., 2021). Wen et al. gathered metabolites data from licorice tablets, stir-fried licorice, and honey-roasted licorice using UPLC-Q-TOF-MS, leveraging multivariate statistical analysis to identify distinguishing components across different licorice processing techniques. A total of 10 quality disparity markers were identified, serving as references for quality control enhancements and processing method optimizations for licorice products (Wen et al., 2020). He et al. identified the active ingredients in Jigucao capsules (JGCC) and the blood prototype metabolites under the condition of the obvious drug effects of JGCC.They revealed a total of 43 prototype blood components and 33 metabolites in JGCC. Quality markers such as abrine, trigonelline, hypaphorine and isoschaftoside were discovered. The established UPLC-MS/MS quantitative analysis method has high sensitivity and accuracy, and can be used for the quality evaluation of JGCC (He et al., 2023). Liu et al. conducted a comparative non-targeted metabolomics analysis of the composition to identify potential markers, enabling qualitative and quantitative evaluation of the quality and optimal harvest period of Aurantii Fructus. Overall, 155 compounds were identified in Aurantii Fructus, with Huangpi exhibiting the highest number of components. 11 potential markers were selected to assess the quality of Aurantii Fructus. The best harvest period of Aurantii Fructus was within 1 week before and after the Lesser Heat (Liu Z. X. et al., 2024).

#### 4.2.3 Application of metabolomics in mechanism of action of TCM

Metabolomics applied to potentially active extracts offers insights into their metabolic pathways, uncovers novel targets and mechanisms of action, and furnishes a foundation for the development of new drugs. Yi et al. found that the mechanism of action of Xuefu Zhuyu Tang in treating coronary heart disease may be related to the processes of bile acid metabolism, lipid metabolism, and linoleic acid metabolism (Yi et al., 2019). Wen et al. enrolled 27 patients who took Chaihu-Guizhi-Ganjiang decoction (CGGD) for 28 days and 30 healthy volunteers as the controls. They verified the effectiveness and explored the mechanism of CGGD in the treatment of chronic non-atrophic gastritis (CNAG) with gallbladder heat and spleen cold syndrome (GHSC) by metabolomics based on UHPLC-Q-TOF/MS.The result suggested that CNAG with GHSC patients were associated with disturbed branched-chain amino acid metabolism and glycerophospholipid levels (Wen et al., 2024). Tang et al. analyzed differential metabolites expression in 70 participants after supplementing with Huoxian Suling Shuanghua Detection (HSSD). The results indicated that the mechanism by which HSSD enhances the immunogenicity of CoronaVac SARS-CoV-2 (CVS) vaccine is related to differential regulation of purine metabolism, vitamin B6 metabolism, folate biosynthesis, arginine and proline metabolism, and steroid hormone biosynthesis (Tang et al., 2024).

### 4.3 Research ideas of metabolomics in TCM

Metabolomics serves as a pivotal tool in unraveling the therapeutic effects of TCM, assessing how variances in varieties and sourcing influence its quality, understanding the mechanisms of action, and identifying potential targets. The research on metabolomics of TCM can be approached from the following points: (1) Metabolomics facilitates the identification of unique metabolite profiles among diverse origins and varieties of TCM. Using multivariate statistical analysis aids in effectively discerning and categorizing these distinctions. (2) The processing of TCM inevitably triggers alterations in its chemical composition. Metabolomics, coupled with data mining techniques, enables a comprehensive examination of substances influenced by processing. Identifying substances exhibiting significant changes in abundance post-processing may underpin efforts to mitigate toxicity and enhance efficiency. (3) The quality of TCM intricately connects with the species and content of plant metabolites. Metabolomics provides a high-throughput method for determining plant metabolites, objectively reflecting differences in chemical composition and medicinal efficacy within TCM. (4) Metabolomics analysis of active or toxic TCM extracts aids in clarifying the metabolic pathways associated with these components. This exploration unveils novel targets and pathways of action, offering valuable insights for new drug development. (5) Comparing metabolic profiles across disease models, control groups, and drug administration groups using metabolomics allows for the pinpointing of differential metabolites. Advanced data analyses, including bioinformatics, chemometrics, and correlation studies, help elucidate the intricate relationships between these markers and biological functions or phenotypes. These insights assist in characterizing changes in related metabolites during drug treatment and their association with disease indicators, aiding in the discovery of metabolites and potential metabolic pathways relevant to their effects (Tang et al., 2021). Using metabolomics to investigate the mechanisms and potential targets of TCM provides essential theoretical foundations for advancing the development and clinical application of new drugs.

## 5 Mass spectrometry imaging in the study of TCM

MSI is an imaging-based ion scanning technology, which can transform MS data into visualized ion imaging maps. It enables the simultaneous *in situ* visualization analysis of multiple molecules, providing insights into metabolite changes across different time and space dimensions. Thus, the high correlation between metabolites and tissue morphology has the advantages of simple preprocessing, clear and intuitive results (Luo et al., 2014). Three primary MSI technologies are commonly utilized: secondary ion mass spectrometry (SIMS), desorption electrospray ionization mass spectrometry (DESI), and matrix-assisted laser desorption ionization (MALDI). SIMS and DESI are mainly suitable for the analysis of small molecule compounds, while MALDI has advantages in macromolecular analysis (Huang et al., 2022a; Huang et al., 2022b; Vickerman, 2011; Liu et al., 2021; Nguyen et al., 2018). These technologies offer distinct capabilities and applications, catering to varied research needs within the field of TCM. For a detailed comparison of the practical applications of the three MSI technologies, refer to Table 3 (Zavalin et al., 2015; Campbell et al., 2012; Wiangnon and Cramer, 2015; Yang and Caprioli, 2011; Wang et al., 2015; Buchberger et al., 2018).

**TABLE 3:** Differences in practical applications of SIMS-MSI, DESI-MSI and MALDI-MSI.

	Suitable molecular weight	Spatial resolution	Sample pretreatment	Collection environment
SIMS-MSI	Small molecule compounds < 3000 Da	100 nm	No need to deposit matrix on the surface of the sample, and can directly conduct research on tissue slices	Vacuum
DESI-MSI	Organic acids, alkaloids, and easily ionized small molecule compounds at 50–2000 Da	50–200 μm	No need to deposit matrix on the surface of the sample, and can directly conduct research on tissue slices	Atmospheric pressure
MALDI-MSI	Small molecule compounds or protein < 200 kDa	20–30 μm	The tested substance and matrix may crystallize uniformly	Vacuum or atmospheric pressure

### 5.1 Workflow of MSI in TCM

**FIGURE 3:**
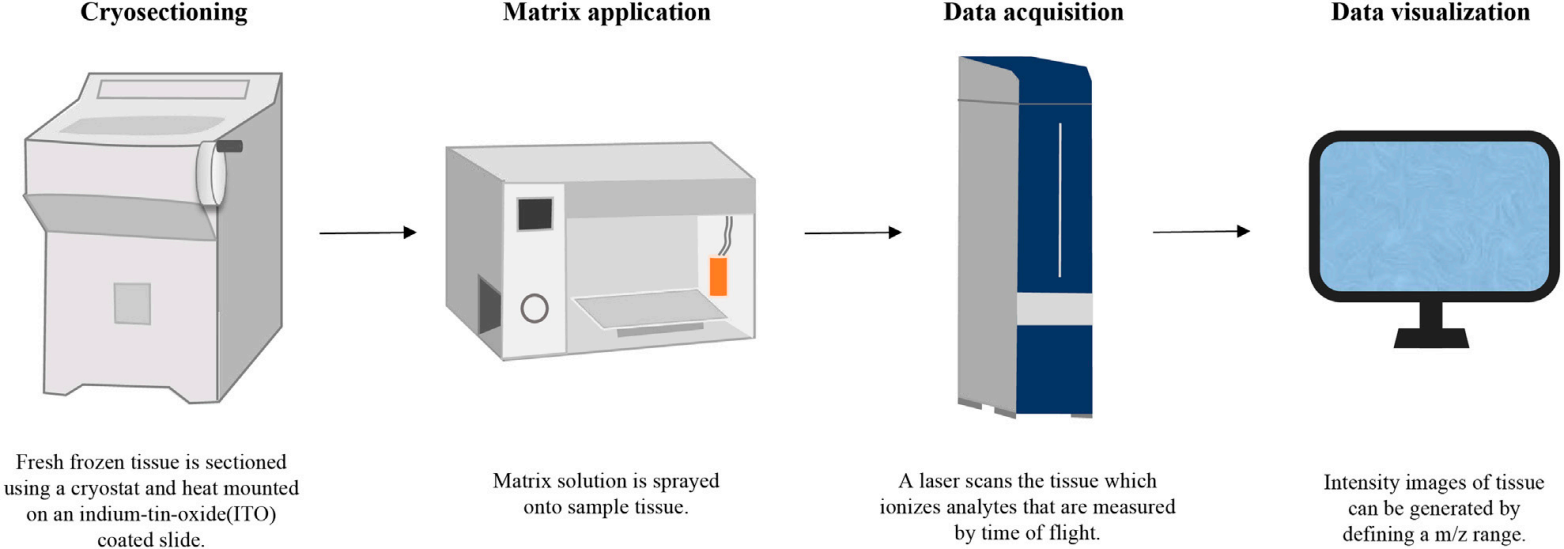
The workflow of MALDI-MSI.

Taking MALDI-MSI as an example, the workflow is shown in Figure 3.

#### 5.1.1 Sample pretreatment

The pretreatment of medicinal materials containing hard roots and rhizomes typically involves four key steps: cutting, embedding, slicing, and pasting. Initially, the medicinal material is segmented into small pieces of specified lengths using a blade. These cut segments are then embedded in suitable media like gelatin or agar. Subsequently, the embedded sample is frozen at low temperatures, and tissue slices are acquired using a cryostat microtome. Finally, these tissue slices are transferred onto a conductive glass plate surface through methods such as hot melt or double-sided conductive adhesive (Li et al., 2020; Lange et al., 2017; Bai et al., 2016; Srimany et al., 2016; Li B. et al., 2014). For medicinal materials characterized by soft textures and uneven surfaces, the transfer printing method is often used for indirect analysis, which not only preserves the spatial distribution information of natural products more completely, but also improves the repeatability of the experiment. In this method, fresh plant tissue is placed on a flat surface material such as a polymer film or silicone plate, and mechanically or manually compressed uniformly. This process facilitates the transfer of components from the plant tissue to the flat surface, allowing for imaging and analysis of the material’s surface with the transferred natural components (Mohana Kumara et al., 2019; Thunig et al., 2011).

#### 5.1.2 Capture optical images

Before collecting MSI data, optical images of tissue slices must be acquired. While most MSI instruments come equipped with micro cameras capable of capturing optical images, the resolution may be limited. Therefore, higher magnification microscopes or scanning electron microscopes are typically utilized to obtain clearer optical images (Li et al., 2016; Fowble et al., 2017).

#### 5.1.3 Spraying substrate

In MALDI-MSI, after collecting optical images, the substrate requires coating for MSI analysis. During matrix selection, classic small molecule matrices such as 2,5-dihydroxybenzoic acid (DHB), α-Cyano-4-hydroxycinnamic acid (CHCA), 9-aminoacidine (9AA) are commonly used for TCM components. It is crucial to be vigilant in excluding interference from matrix peaks during subsequent data processing to ensure accurate results (Qiao and Lissel, 2021). On the other hand, in DESI-MSI and SIMS-MSI, data collection can commence directly after obtaining optical images, eliminating the need for substrate coating.

#### 5.1.4 Data collection and processing

After collecting MS data at various coordinate points on the surface of the sample under appropriate experimental conditions, a substantial amount of data need to be imported into the computer. These raw data are processed and reconstructed through software tools to generate imaging maps for each target ion (Sturtevant et al., 2016). By comparing the previously collected optical images with obtained MSI images, the specific distribution information of a certain component can be located in detail. For a more comprehensive analysis, further processing of MSI data combined with multivariate analysis techniques can yield deeper insights based on visual examination (Huang et al., 2022a; Huang et al., 2022b; Ràfols et al., 2018). This integrated approach not only enhances the interpretation of complex data sets but also allows for the extraction of more detailed and meaningful information regarding the spatial distribution and composition of key components in TCM samples.

### 5.2 Application of MSI in TCM

In recent years, MSI has been applied to reveal the spatial distribution characteristics of natural components, explore the pharmacological mechanisms, and research new methods for evaluating the quality, bringing a new perspective to TCM research (Huang et al., 2022b). Here, we summarize MSI studies of TCM and highlight the application of MSI in spatial distribution of natural components and pharmacological mechanism of TCM, as shown in Table 4.

**TABLE 4:** The application of MSI in spatial distribution of natural components and pharmacological mechanism of TCM.

Application	Study findings	Reference
Spatial distribution of natural components	Distribution characteristics of gallic acid tannins and their intermediates in the root of Paeonia lactiflora were reported	Li et al. (2021)
	Spatial distribution of natural components in Isatis indigotica in both positive and negative ion mode was comprehensively visualized through microscopic MSI	Nie et al. (2021)
	Systematic characterization and spatial tissue distribution studies of ginsenosides in the rhizome of Panax japonicus var. major (PJM) was reported	Jiang et al. (2023)
	Spatial distribution of natural components in Polygonum multiflorum, Curcuma longa, Paris polyphylla var. yunnanensis, Panax ginseng, Panax notoginseng, Aquilaria sinensis, Morus alba Linn, etc, were explored	Zhao et al. (2021), Shimma and Sagawa (2019), Kuo et al. (2019), Sun et al. (2021)
Pharmacological mechanism	Pharmacological mechanisms through which aconite extract mitigates myocardial injury was shed light	Wu et al. (2019)
	The mechanism underlying the herbal combination consisting of RS in a rodent model for AD might intricately linked to neuroinflammation, neurotransmitter imbalance, energy deficiency, oxidative stress, and aberrant fatty acid metabolism	Fan et al. (2024)

#### 5.2.1 Application of MSI in spatial distribution of natural components of TCM

At present, MSI has emerged as a valuable tool for unveiling the distribution patterns of essential medicinal components within plant tissues. These findings can guide the extraction of target components from specific distribution areas, thereby enhancing the efficiency of natural component enrichment processes and facilitating the optimal utilization of TCM resources (Huang et al., 2022a). Li et al. used MALDI-MSI to investigate the distribution characteristics of gallic acid tannins and their intermediates in the root of Paeonia lactiflora, offering insights into synthesis mechanisms (Li et al., 2016; Li et al., 2021). Nei et al. utilized atmospheric pressure matrix-assisted laser desorption ionization (AP-MALDI) and ion trap time of flight mass spectrometry (IT-TOF/MS) to comprehensively visualize the spatial distribution of natural components in Isatis indigotica in both positive and negative ion modes through microscopic MSI (Nie et al., 2021). Jiang et al. integrated UHPLC/QTOF-MS and DESI-MSI for the systematic characterization and spatial tissue distribution studies of ginsenosides in the rhizome of Panax japonicus var. major (PJM). They achieved comprehensive characterization of the ginsenosides in the rhizome of PJM with a large amount of unknown structures unveiled primarily and reported the spatial tissue distribution of different subtypes of ginsenosides in the rhizome slice of PJM (Jiang et al., 2023). Furthermore, MSI has been instrumental in investigating the spatial distribution of natural components in various medicinal materials such as Polygonum multiflorum, Curcuma longa, Paris polyphylla var. yunnanensis, Panax ginseng, Panax notoginseng, Aquilaria sinensis, Morus alba Linn, etc (Zhao et al., 2021; Shimma and Sagawa, 2019; Kuo et al., 2019; Sun et al., 2021). These studies collectively highlight the diverse applications of MSI in characterizing the spatial distribution of bioactive compounds within medicinal plants, contributing significantly to the advancement of TCM research and practice.

#### 5.2.2 Application of MSI in pharmacological mechanism of TCM

The integration of MSI has provided a new perspective for the research of TCM pharmacology. Currently, MSI is being increasingly utilized to visually illustrate the alterations in metabolite responses within animal tissues post-administration of active substance monomers, extracts, and TCM preparations (Huang et al., 2022a). This innovative approach aids in unraveling the therapeutic mechanisms of various TCM treatments. For instance, Wu et al. established a rat model and used MALDI-MSI to visualize heart tissue following the administration of aconite extract. This study shed light on the pharmacological mechanisms through which aconite extract mitigates myocardial injury, showcasing the versatility and effectiveness of MSI in exploring the intricate interactions between TCM formulations and biological systems (Wu et al., 2019). Fan et al. used air flow-assisted desorption electrospray ionization mass spectrometry imaging (AFADESI-MSI) and network pharmacology to investigate the pharmacodynamics and mechanism underlying the herbal combination consisting of Radix ginseng-Schisandra chinensis (RS) in a rodent model for Alzheimer’s disease (AD). 28 biomarkers were identified, which are intricately linked to neuroinflammation, neurotransmitter imbalance, energy deficiency, oxidative stress, and aberrant fatty acid metabolism in AD. They also constructed a target interaction network based on the corresponding bioactivities that revealed relationships amongst 11 key biomarkers, 8 active ingredients and 12 critical targets (Fan et al., 2024).These research endeavors underscore the importance of MSI as a powerful tool for investigating the intricate pharmacological effects of TCM interventions at a molecular level, offering valuable insights into their therapeutic actions and underlying mechanisms.

### 5.3 Research ideas of MSI in TCM

MSI in TCM is primarily focusing on investigating the spatial distribution and content disparities of active ingredients within various medicinal materials. This analytical technique aids in elucidating the synthesis mechanisms of medicinal components and provides valuable insights into the action mechanisms of TCM formulations. To advance MSI research in TCM, the following points can serve as a reference: (1) Revealing the accumulation sites of TCM ingredients in different plant tissues, providing basis for exploring the biosynthetic mechanism of medicinal ingredients and improving the extraction process of TCM. (2) Combined with multivariate analysis, in-depth exploration of MSI data can screen out indicator components with practical significance and guide the identification of medicinal materials. (3) The *in situ* analysis of medicinal ingredients, as well as the study of the distribution sites of impurities, allergenic components, and harmful substances, bring new thought to drug safety research. (4) To provide a new perspective for the research of TCM pharmacology, MSI can be used to visually present the changes in metabolites of animal tissues post-administration of TCM, and assist in exploring the mechanism of action of TCM (Huang et al., 2022b).

## 6 Combination of multi-omics in TCM

In multidisciplinary biology, omics methods are defined as high-throughput, information rich assays to obtain a set of molecular measurement results within cells or tissues. A comprehensive understanding of human health and diseases requires explaining the complexity and variability of molecules at multiple levels, and the availability of multi-omics data has revolutionized the fields of medicine and biology (Yan et al., 2018). Genes, transcripts, proteins, metabolites, and other macro/micro molecules collaborate systematically to execute complex cellular processes. Integrating multi-omics data and providing information on biomolecules from different layers seems promising for a systematic and comprehensive understanding of complex biology (Bersanelli et al., 2016). It has been widely shown that the integration of multi-omics can help elucidate potential mechanisms at multiple omics levels. Integrated methods, with their ability to comprehensively study biological phenomena, have the ability to improve the prognosis and prediction accuracy of disease phenotypes, ultimately contributing to better treatment and prevention (Hasin et al., 2017).

The common approach of multi-omics integration analysis is to screen target biomolecules, analyze the functions of target molecules based on the functional hierarchy logic of systems biology, and integrate multi-omics data based on collaborative network regulation logic (Subramanian et al., 2020). Through data integration and analysis, mutual verification and supplementation, a comprehensive understanding of the trends and directions of biological changes is ultimately achieved, a molecular biology change mechanism model is proposed, and key metabolic pathways or genes, transcripts, proteins, metabolites are screened for further in-depth experimental analysis and application (Kim et al., 2016). The disease status and changes after drug treatment obtained through genomics, transcriptomics, proteomics, and metabolomics experiments can enrich and trace the pathways with the greatest and most concentrated changes. Through comprehensive analysis of genes, mRNA, proteins, and small molecules in the body, including the analysis of original pathways and the construction of new pathways, cellular function and metabolic status are reflected, providing new ideas and methods for exploring biological regulatory molecular mechanisms, key biomarkers, and targets (Fondi and Liò, 2015).

**FIGURE 4:**
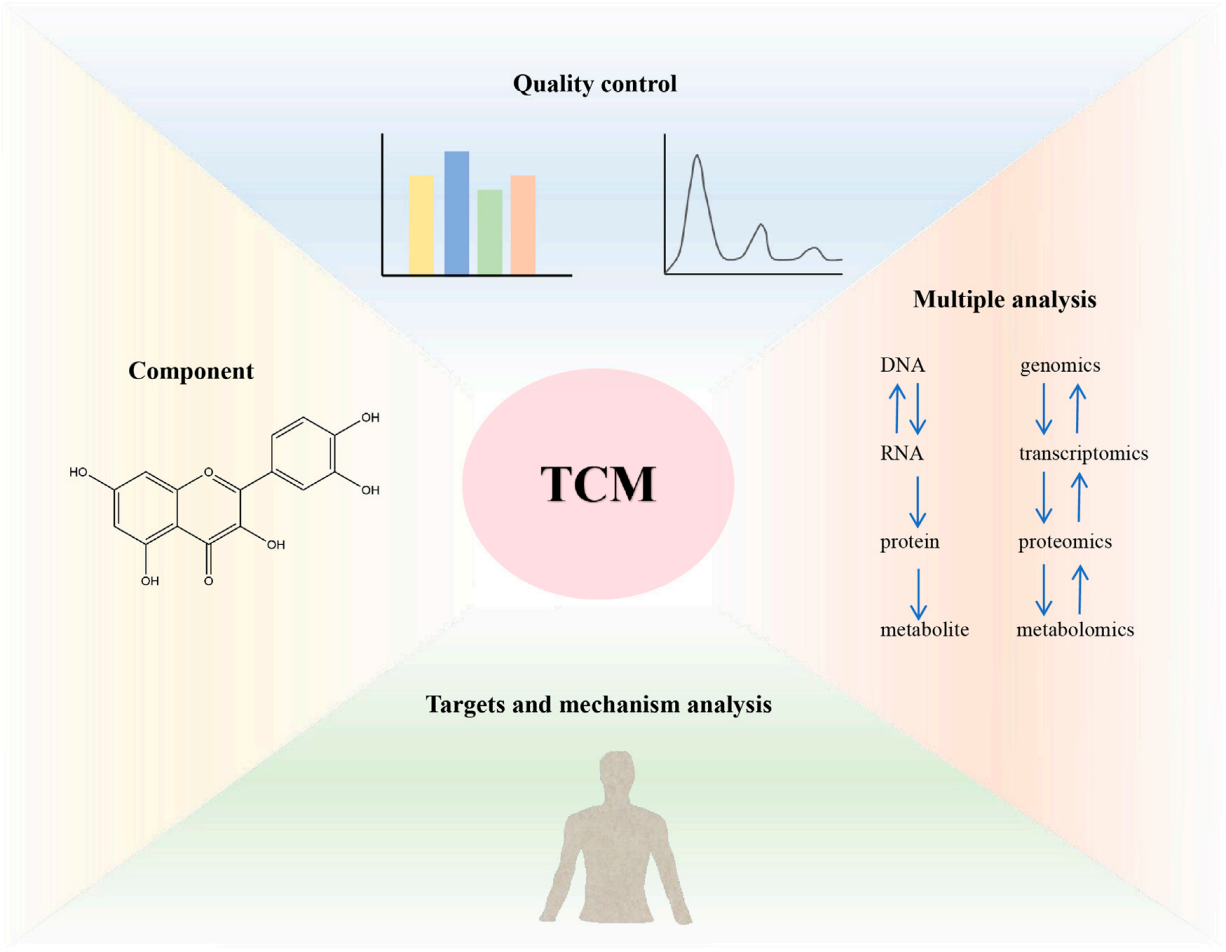
The combination of multi-omics promotes the research of TCM.

The high-throughput omics methods at different levels have helped TCM research solve problems in modern society, achieving rapid and cost-effective component identification, quality control, targets analysis, molecular mechanisms, drug toxicity, and clinical validation (Buriani et al., 2012). The strategy of combining multi-omics to research TCM is shown in Figure 4. In recent years, the advancement of systems biology and clinical personalized medicine has driven a paradigm shift from single target specificity to a holistic view of biological systems with dynamic complexity. By integrating omics data and powerful analytical strategies such as bioinformatics and computational tools, TCM research has expanded its focus from the perspective of “one target, one drug” to the perspective of “network targets, multi-component therapy”. Therefore, multi-omics has been rapidly applied to almost all biomedical fields, including TCM, and is crucial for understanding the biological basis of TCM symptoms and providing molecular evidence for creating new TCM therapeutic drugs (Li S. et al., 2014; Feng et al., 2022). Given the widely recognized role of TCM in modern medicine, the emergence of multi-omics platforms can help TCM meet the precision medicine requirements proposed by Western medicine and gain new insights into TCM (Zhang et al., 2023; Guo et al., 2020).

In recent years, various studies have shown that combining omics datasets can provide a better and clearer understanding of the system under study. Zhao et al. used multi-omics to investigate the regulatory effect of andrographolide (Andro) on neuroinflammation in VCI patients. Their result suggested that in the VCI model, Andro may alleviate neuroinflammation, neuronal damage, and cognitive impairment. Andro blocks the mitogen activated protein kinase (MAPK) pathway to manage neuroinflammation in VCI, ultimately restoring neuronal damage and cognitive impairment. Therefore, the MAPK pathway has become an important target pathway for Andro’s treatment of VCI (Zhao et al., 2022). Wang et al. explored the protective mechanism of geniposide on damaged liver tissue through proteomics and metabolomics. 9 differential endogenous metabolites were identified, which are related to primary bile acid biosynthesis, butyric acid metabolism, citric acid cycle, alanine, aspartic acid, and glutamate metabolism disorders. At the same time, a protein interaction network was constructed to help identify the drug targets of geniposide. 6 identified differential proteins are involved in antioxidant, signal transduction, energy production, immunity, metabolism, and accompanying effects. These proteins are closely related to the regulation of PPI networks and various important physiological pathways (Wang et al., 2013b). Zhao et al. integrated computational analysis including network target analysis and machine learning algorithms with clinical multi-omics experiments and public omics data to explore the machanism of Tuomin Zhiti Decoction (TZD) in treating seasonal allergic rhinitis (SAR). They identified the potential impact of TZD on immune response and downstream immune cells in SAR therapy through direct mechanisms involving antigens and indirect mechanisms mediated by gut microbiota (Zhao et al., 2024). These studies widely demonstrate the importance of integrating multi-omics over single omics analysis.

Moreover, with the rapid development of science and technology, artificial intelligence (AI) has been intertwined with many aspects of life. Due to the advancement of computer data processing capabilities and the rapid accumulation of large datasets, more accurate drug target inference algorithms have been developed (Zhang et al., 2023). The combination of AI and multi-omics is now an effective method for discovering new drugs. The clinical stage of drug development is significantly influenced by AI such as machine learning, compound to small molecule structure docking, compound characteristic dynamics simulation, computer-aided drug synthesis, new molecule design, and target protein structure prediction (Lv et al., 2024). Based on the molecular pathways discovered during the material screening process, machine learning is used to establish a predictive model for molecular features of compounds and identify TCM evidence. In addition, the rapid development of AI will provide considerable technical and algorithmic support for identifying new drugs. The construction of supervised and unsupervised multidimensional mathematical models will achieve to discover ideal drug therapeutic doses and unique components more accurately and scientifically (Chan et al., 2019). For example, artificial neural networks, a deep learning method, can be used to stratify the relationship between drug dosage and treatment efficacy in preclinical animal studies (Oladipo et al., 2022). High specificity and intelligence analysis methods may significantly reduce the required physical labor and improve the success rate of clinical trials. Combining AI with multi-omics is expected to make new progress in TCM research.

## 7 Conclusion and viewpoint

TCM represents a quintessential complex material system, presenting a dual nature of complexity that serves as both a strength and a challenge. The diversity of its components makes it more systematic in treating diseases, however, the complexity of its components also makes the study of mechanism very difficult. To address these challenges, it is imperative to incorporate cutting-edge technologies and evolving scientific methodologies into the study of TCM complex systems, truly revealing the material basis and treatment mechanism of action of TCM. Therefore, TCM research can truly move towards modernization.

In the era of big data, systems biology based on multi-omics platforms is an essential tool for the modernization of TCM research. The application of multi-omics to investigate the complexities of TCM systems has yielded novel insights and methodological frameworks for advancing the modernization of TCM practices. The advent of sequencing technologies has ushered in a new era in medical and biological research, where omics-based data play a pivotal role in understanding intricate biological processes. The collective utilization of multi-omics has revolutionized medicine and biology, offering unprecedented means to dissect the mechanisms by which TCM treats various diseases. However, standardizing multi-omics to obtain reliable results is essential. It includes standardization of sample collection and processing (following unified operating procedures), standardization of experimental design (ensuring consistency and reproducibility of experimental conditions), standardization of data analysis processes (using consistent analysis methods and parameters), quality control (strict quality monitoring of experimental processes and data), reference standards and control settings (using appropriate standards and control samples), and data sharing and validation (comparing and validating with other studies). We need to standardize omics research and continuously improve standards in the future.

Delving into the mechanistic underpinnings of TCM represents a crucial trajectory for the future evolution of TCM on the global stage. With technological advancements continuing unabated, the journey towards the worldwide modernization of TCM appears imminent. As we stride towards this transformative future, the fusion of innovative methodologies and interdisciplinary collaborations will undoubtedly propel TCM research into a new era of scientific discovery and clinical efficacy.
