# Supplementary material for: Application of multi-omics in the study of traditional Chinese medicine
Source: Front Pharmacol. 2024 Sep 6;15:1431862. doi: 10.3389/fphar.2024.1431862 (PMC11412821; doi:10.3389/fphar.2024.1431862)
Supplement: Supplementary file 1 [file Table1.DOCX]

**Supplementary table 1 The advantages and disadvantages of multi-omics.**

|  | **Advantages** | **Disadvantages** |
| --- | --- | --- |
| **Genomics** | - identify TCM resources - analysis genetic diversity | - requires high technical and professional knowledge to analyze and interpret data |
| **Transcriptomics** | - understand the changes in gene expression under the action of TCM | - complex data may affect the accurate understanding of results |
| **Proteomics** | - Identify protein from unique peptides comprising the protein. - predictable fragmentation patterns - no require standard reference proteins for protein assignments - reveal targets and regulatory mechanisms of TCM | - dynamic changes and complexity of proteins may make analysis difficult - slow protein expression profile dynamics - sample preparation is often multi-step |
| **Metabolomics** | - common metabolites across species - shared experimental evidence to guide identifications - fast metabolic dynamics - simple or complex sample preparation depending on goals of the experiment. - identify potential biomarkers | - lack of standard reference material for many metabolites - metabolite identification cannot be inferred from fragments comprising the whole metabolite - similar fragments for different species |

**Supplementary table 2 Advantages and problems of MS, NMR, LC, GC, CE, UV-Vis, FTIR.**

|  | **Advantages** | **Problems** |
| --- | --- | --- |
| **MS** | - Realize identification and quantification of volatile and thermally stable components - Used for ionization of polar to nonpolar components | - Being destructive - Requiring pre-knowledge about samples - High recurrent expenditures |
| **NMR** | - Noninvasive and nondestructive for samples - Quantitative and simultaneous detection unbiased for any molecules - High throughput - Produces rich, dynamic molecular information - Requires little or no sample preparation - Good resolution and reproducibility | - Low sensitivity - Low concentration components are not easily detected |
| **LC** | - Low cost - Easy to use - Highly sensitive - Not limited by sample volatility and stability - Favorable separating power | - Almost exclusively used for target component analysis and not for whole-sample fingerprinting combined with chemometrics |
| **GC** | - Highly sensitive detection for almost both volatile chemical and nonvolatile compounds - Has more peak capacity and can accommodate more complex mixtures | - Unsuitable for nonvolatile and thermally unstable compounds |
| **CE** | - High speed and short analysis time - Less sample and solvent consumption - Appropriate for complex samples - Lower operating cost | - Lower sensitivity |
| **UV-Vis** | - Easy to be applied | - Limited use for compounds without UV absorption - Lacks specificity |
| **FTIR** | - Potential to use vibrational spectrometry | - Lacks specificity - Signal overlapping |

**Supplementary table 3 Confidence annotation and statistical evaluation approach of metabolite identification.**

| **Identification confidence** | **Annotation** | **Utility** | **Statistical treatment/**  **Approach** |
| --- | --- | --- | --- |
| **Level 1** | confirms a structure with a minimum of two orthogonal properties from a pure reference standard acquired under identical analytical conditions | Integration with known biology | Pathway and network  analyses |
| **Level 2** | a lack of reference standard acquisition but predictive or externally acquired structure evidence, namely MS/MS data, exhibiting diagnostic fragments or neutral losses consistent with a specific structure would be considered a putative identification |  |  |
| **Level 3** | arise when accurate mass and isotopic distribution pat  terns produce tentative structures from database searches | Matching parent ion exact mass and fragmentation patterns | MS1 database and MS2 spectral library |
| **Level 4** | complete the less confident annotation classifications | Ranking significant differences  Data visualization and prioritization  Pathway/network analysis without formal annotation | t-test  Principle Component Analysis (PCA)  Partial Least Squares (PLS) Modeling  Cloud Plot/Volcano Plot Self-organizing Map (SOM)  Pathway and network prediction |
| **Level 5** |  |  |  |
